# Assessment of the prognostic value of preoperative high-sensitive troponin T for myocardial injury and long-term mortality for groups at high risk for cardiovascular events following noncardiac surgery: a retrospective cohort study

**DOI:** 10.3389/fmed.2023.1135786

**Published:** 2023-06-22

**Authors:** Yingchao Zhu, Yaodan Bi, Qian Yu, Bin Liu

**Affiliations:** ^1^Department of Anesthesiology, West China Hospital, Sichuan University, Chengdu, Sichuan, China; ^2^Department of Anesthesiology, Peking Union Medical College Hospital, Peking Union Medical College and Chinese Academy of Medical Sciences, Beijing, China; ^3^Department of Anesthesiology, Public Health Clinical Center of Chengdu, Chengdu, Sichuan, China

**Keywords:** risk assessment, serum biomarkers, preoperative myocardial injury, high-sensitivity cardiac troponin, non-cardiac surgery, postoperative outcomes, mortality, mediation analysis

## Abstract

**Background:**

Few studies explored the association between high-sensitive cardiac troponin T (hs-cTnT) and long-term mortality for patients after surgery. This study was conducted to assess the association of hs-cTnT with long-term mortality and to investigate the extent to which this association is mediated via myocardial injury after noncardiac surgery (MINS).

**Methods:**

This retrospective cohort study included all patients with hs-cTnT measurements who underwent non-cardiac surgery at Sichuan University West China Hospital. Data were collected from February 2018 and November 2020, with follow-up through February 2022. The primary outcome was all-cause mortality within 1 year. As secondary outcomes, MINS, length of hospital stay (LOS), and ICU admission were analyzed.

**Results:**

The cohort included 7,156 patients (4,299 [60.1%] men; 61.0 [49.0–71.0] years). Among 7,156 patients, there were 2,151 (30.05%) with elevated hs-cTnT(>14 ng/L). After more than 1 year of follow-up, more than 91.8% of mortality information was available. During one-year follow-up after surgery, there were 308 deaths (14.8%) with a preoperative hs-cTnT >14 ng/L, compared with 192 deaths (3.9%) with a preoperative hs-cTnT <=14 ng/L(adjusted hazard ratio [aHR] 1.93, 95% CI 1.58–2.36; *p* < 0.001). Elevated preoperative hs-cTnT was also associated with several other adverse outcomes (MINS: adjusted odds ratio [aOR] 3.01; 95% CI, 2.46–3.69; *p* < 0.001; LOS: aOR 1.48, 95%CI 1.34–1.641; *p* < 0.001; ICU admission: aOR 1.52, 95%CI 1.31–1.76; *p* < 0.001). MINS explained approximately 33.6% of the variance in mortality due to preoperative hs-cTnT levels.

**Conclusion:**

Preoperative elevated hs-cTnT concentrations have a significant association with long-term mortality after noncardiac surgery, one-third of which may by accounted for by MINS.

## Introduction

According to research, more than 300 million non-cardiac operations are performed worldwide each year (1). The incidence of perioperative cardiovascular complications is between 3.6 and 7% (2), which is an important source of perioperative morbidity and mortality, as well as an important cause of prolonged hospitalization and increased medical burden (3). The risk of perioperative adverse cardiac events was even higher in patients with confirmed or underlying coronary artery disease (4, 5). Cardiac troponin I and T (cTnI/T) are myocardial-specific proteins released into the blood by cardiomyocytes following injury to the myocardium (6). The prognostic value of postoperative cTn has previously been investigated by large multicenter prospective studies (7, 8). In contrast, the value of preoperative and perioperative changes in cTn as a prognostic tool for adverse outcomes has been sparsely investigated.

Meta-analyses (9, 10) had demonstrated an association between preoperative TnT levels and short-term major adverse cardiovascular events (MACE) and mortality, but studies on long-term adverse outcomes are limited. Several studies (11–14) have performed univariate analyses of preoperative cTn and long-term adverse outcomes. However, the total sample size was less than 600, and there was substantial heterogeneity among the studies. Only two studies (3, 15) have investigated adjusted associations. Although the two studies obtained similar results, Hietala et al. (15) did not use high sensitive cTnT and only included hip fracture surgery. Meanwhile, Nagele et al. (3) did not adjust for important potential confounders, e.g., the RCRI score (16–18) and intraoperative hypotension (19, 20), reducing the reliability in assessing the independent prognostic value of preoperative cTn. With the popularization of hypersensitive troponin test methods, more and more patients are undergoing the hs-cTnT test before operation (11, 21–23). Therefore, the relationship between preoperative hs-cTnT level and long-term outcomes still needs more careful correlation analysis. In addition, the extent to which preoperative and perioperative changes in cTn [e.g., myocardial injury after noncardiac surgery (MINS)] mediate the relationship between preoperative hs-cTnT and postoperative mortality has not been demonstrated.

We aimed to determine the associations between preoperative hs-cTnT levels and long-term outcomes by multivariable Cox regression models and to explore the mediating effect of MINS by mediation analysis.

## Methods

### Study design and data collection

This is a retrospective cohort study that adheres to the Strengthening the Reporting of Observational Studies in Epidemiology (STROBE) (24) statement. Most of the data were extracted from Hospital Information System (HIS) and Anesthesia Information Management System (AIMS). In-hospital deaths were documented with death certificates and medical record reviews, and out-of-hospital deaths and dates of death were obtained through telephone follow-up. Because of the sensitive nature of the data collected for this study, data collection was performed by unwitting hospital information center staff. After extracting the raw data of the preoperative evaluation sheets, the baseline characteristics were organized into a standardized form by independent investigators who were blinded to the outcomes. Data analysis was performed by qualified researchers trained in human subject confidentiality agreements. For purposes of confidentiality, all data were de-identified and analyzed anonymously. Ethical approval was obtained from the Ethics Committee of Sichuan University (Project No. 1082 in 2021). The study was registered at chictr.org (ChiCTR2200058376).

We identified consecutive patients who underwent surgery between February 2018 and November 2020 at West China Hospital, Sichuan University. Inclusion criteria: (1) Age > 16 years; (2) Patients who underwent non-cardiac surgery; (3) Patients who underwent hs-cTnT examination within 7 days before surgery; (4) ASA Grade I-V. Exclusion criteria: (1) Patients undergoing diagnostic, cardiac, obstetric, and ophthalmic surgery; (2) Hospital stay less than 24 h (day surgery).

### Outcomes and variables

The primary outcome measure was defined as all-cause mortality 1 year after surgery. The secondary outcome measures were defined as MINS, length of hospital stay (LOS), and postoperative ICU admission. MINS (8) was defined as the concentration of hs-cTnT ≥20 ng/L and the absolute value change ≥5 ng/L, or the absolute value of hs-cTnT ≥65 ng/L, and no such detection was considered as no postoperative myocardial injury. Peak postoperative values regardless of the day of sampling were used in these calculations.

We also generated a list of risk-adjustment variables (Supplementary Table 1), including patient age, sex, body mass index (BMI), 15 preoperative comorbidities, 12 preoperative laboratory tests, 5 prognostic models (ASA, CCI, RCRI, Ex-care, SORT), types of surgery, and detail of anesthesia and intraoperative management. Preoperative anemia was defined as hemoglobin <130 g /L for men and < 120 g/L for women, preoperative increased creatinine was defined as plasma levels of creatinine 100 mmol/L for men and 90 mmol/L for women, intraoperative transfusion was defined as intraoperative transfusion of any blood product, and intraoperative hypotension was defined as MAP<55 mmHg at any time intraoperatively (regardless of duration).

### Hs-cTnT measurements and management

Serum TnT examination is not a routine preoperative test for surgical patients in our institute. The clinicians screened high-risk groups to detect myocardial injury according to clinical guidelines and experience. We defined the preoperative hs-cTnT level as the serum concentration of the last hs-cTnT measurement within 7 days before surgery. We obtained fresh serum aliquots from supernatants of peripheral or central blood samples collected in lithium heparinized tubes that were previously centrifuged for 5 min at 3500× *g* in our Core Laboratory. The levels of serum hs-cTnT were measured by Elecsys Troponin T hs of non-competitive enzyme-linked immunosorbent assays (ELISA) using a Cobas e602Modular AutoAnalyzer (Roche Diagnostics, Basel, Switzerland). The minimum detectable value was 3 ng/L.

### Statistical analysis

We first compared characteristics of patients with or without elevated preoperative hs-cTnT using Fisher exact tests or *χ*^2^ tests for categorical measures and 2-sample t tests or Mann–Whitney *U* tests for continuous measures. Summary statistics are presented as mean (standard deviation), median [inter-quartile range (IQR)], or number (%).

To assess unadjusted associations between different hs-cTnT levels and time to the primary composite outcome, we first used stratified Kaplan–Meier plots with log-rank tests. Hazard ratios (HRs) were then reported using univariate Cox regression models that included only hs-cTnT levels as explanatory variables. We then fitted multivariable Cox regression models, adjusting for patient demographic characteristics, comorbidities, preoperative laboratory tests, hepatic and renal function, and detail of anesthesia and intraoperative managements (25). Univariate analysis of each factor was performed (Supplementary Table 1). Variables were considered candidates for inclusion in the risk-adjustment model if there were bivariable associations with the primary outcome event at *p* < 0.10. We selected final variables for the multivariable Cox model using the both-sided stepwise regression method. Collinearity was evaluated by the variance inflation factor (VIF) (26), and only variables with VIF ≤ 10 were input into the final model. We assessed multivariable Cox model appropriateness by receiver operating curve (ROC) and calibration curve (27, 28). Logistic regression was also used to assess the association of preoperative hs-cTnT and MINS, LOS, and ICU admission reported as odds ratios (ORs) with 95% confidence intervals (CIs). Statistical analyses were performed using R 4.0.2 and R-packages (tableone, survminer, survival, rms, MASS, AUC, calibrate and ggsurvplot).

The sample size required for developing a clinical prediction model is calculated according to the guidance published in 2020 (29). Use pmsampsize to calculate the minimum sample size required for developing a multivariable prediction model with a survival outcome using 50 candidate predictors. We expected all-cause mortality of about 7% after noncardiac surgery, a mean follow-up of more than 200 days, and an adjusted R-squared of 0.19. We select a time point of interest for prediction using the newly developed model of 1 year. The result indicates that at least 3,205 samples are required, corresponding to 1756.2 person-time of follow-up and 2.46 events per covariate. Sample size calculation was performed using R-packages (pmsampsize).

### Subgroup analyses

We conducted subgroup analyses to determine whether associations between hs-cTnT levels and one-year mortality varied according to different ages, BMI, ASA-PS scores, emergency cases, surgical categories, and comorbidities. Due to the minimum sample size limitation, only partial subgroup analyses were performed for the type of surgery. For every risk-factor subgroup, the respective variables defining the risk factor were removed from the analyses. Every subgroup was treated as new data to calculate the HR for the association between the preoperative hs-cTnT level and outcomes. All *p* values were 2-sided, with *p* < 0.05 defined as the threshold of statistical significance. Forestplot of subgroup analyses was performed using R-packages (forestplot).

### Mediation analyses

To investigate the mediation effect (30, 31) of MINS between preoperative hs-cTnT level and one-year mortality, structural equation modeling (SEM) analysis (32) was performed using the lavaan package (33) on the R software and graphically reported using the lavaanPlot package. Two models were estimated: a multivariate logistic regression model for MINS (mediator) conditional on hs-cTnT and all study confounders and a multivariate linear regression model for the mortality (outcome) conditional on hs-cTnT, MINS, and all study confounders. The natural direct effects (NDE) represented the effect of hs-cTnT on mortality that was independent of MINS. The natural indirect effects (NIE) represented the proportion of hs-cTnT that could be explained by its association with MINS. To quantify the magnitude of mediation, the study estimated the proportion of the association mediated by MINS (NIE/[NDE + NIE]). All analyses were estimated using bootstrapping (1,000 replications) to recover the correct standard errors for direct and indirect effects (32). All *p* values were 2-tailed, and statistical significance was set at a *p* value less than.05.

## Results

### Baseline characteristics

A total of 7,156 who underwent hs-cTnT measurements before non-cardiac surgery were enrolled in our trial. Participant flow is outlined in Supplementary Figure 1. In 2151 patients (30.1%), the hs-cTnT concentration was above the 99th percentile of the upper range limit (URL ≥ 14 ng/L). The median preoperative hs-cTnT was 9.20 ng/L (IQR 6.10–23.75). The mean time for hs-cTnT testing before surgery was 2.3 days (SD 1.28).

The characteristics of included patients are summarized in Table 1. A relatively small proportion (ie, 589 [8.23%]) were censored on February 1, 2022, with more than 1 year of follow-up. We identified 617 (9.2%) deaths, 644 (9.0%) MINS, and 2099 (29.3%) ICU admission after surgery during follow-up. The median length of hospital stay was 12.0 days (IQR 8.0–20.0). Patients with higher ASA and RCRI grades, SORT, and Ex-care scores also had higher hs-cTnT levels before surgery. The preoperative hs-cTnT level is correlated with intraoperative hypotension, rapid heart rate, increased blood loss, and transfusion. However, age, gender, BMI, and operation duration had no significant correlation with preoperative hs-cTnT concentration. Although differences in all preoperative laboratory tests were statistically significant in this large data set, most of the differences were generally of small magnitude. Compared with patients with normal preoperative hs-cTnT, those with elevated hs-cTnT were more likely to have more ICU admission (1,286 [25.7%] vs. 813 [62.2%]; *p* < 0.001), more MINS (231 [4.6%] vs. 413 [19.2%]; *p* < 0.001), and a longer median (IQR) stay in hospital (11 [8–18] days vs. 15 [8–24] days; *p* < 0.001). Similarly, preoperative hs-cTnT elevation was significantly associated with 30-day (162 [3.5%] vs. 248 [12.9%]; *p* < 0.001), 90-day (191 [4.1%] vs. 302 [15.8%]; *p* < 0.001) and one-year mortality (192 [3.9%] vs. 308 [14.8%]; p < 0.001).

**Table 1:** Characteristics stratified by hs-cTnT groups.

Variable		Level	Overall	hs-TnT < =14 ng/L	hs-TnT > 14 ng/L	*p* value
*n*			7,156	5,005	2,151	
Sex (n[%])	Men		4,299 (60.1)	2,872 (57.4)	1,427 (66.3)	<0.001
	Women		2,857 (39.9)	2,133 (42.6)	724 (33.7)	
Age (median[IQR])			61.00 [49.00,71.00]	59.00 [49.00,68.00]	66.00 [51.00,76.00]	<0.001
BMI (median[IQR])	BMI		23.00 [21.00,25.00]	23.00 [21.00,25.00]	23.00 [20.0,25.00]	<0.001
Prognostic models	ASA (n[%])	I-II	2,560 (35.8)	2,218 (44.3)	342 (15.9)	<0.001
		III	3,956 (55.3)	2,514 (50.2)	1,442 (67.0)	
		IV-V	640 (8.9)	273 (5.5)	367 (17.1)	
	CCI (n[%])	<=4	5,945 (83.1)	4,165 (83.2)	1780 (82.8)	0.85
		5–8	963 (13.5)	666 (13.3)	297 (13.8)	
		> = 9	248 (3.5)	174 (3.5)	74 (3.4)	
	RCRI (n[%])	0	3,189 (44.6)	2,396 (47.9)	793 (36.9)	<0.001
		1	2,989 (41.8)	2,132 (42.6)	857 (39.8)	
		2	838 (11.7)	430 (8.6)	408 (19.0)	
		> = 3	140 (2.0)	47 (0.9)	93 (4.3)	
	SORT (median[IQR])		1.45 [0.64,2.71]	1.06 [0.04,2.33]	2.27 [0.92,3.22]	<0.001
	Excare (median[IQR])		19.88 [13.22,24.13]	19.88 [13.22,22.58]	22.58 [19.88,25.82]	<0.001
Comorbidities (n[%])	Ischemic heart disease	No	6,465 (90.3)	4,618 (92.3)	1847 (85.9)	<0.001
		Yes	691 (9.7)	387 (7.7)	304 (14.1)	
	Atrial fibrillation	No	6,908 (96.5)	4,916 (98.2)	1992 (92.6)	<0.001
		Yes	248 (3.5)	89 (1.8)	159 (7.4)	
	Chronic heart failure or cardiomyopathy	No	7,092 (99.1)	4,983 (99.6)	2,109 (98.0)	<0.001
		Yes	64 (0.9)	22 (0.4)	42 (2.0)	
	Valvular disease	No	6,869 (96.0)	4,852 (96.9)	2017 (93.8)	<0.001
		Yes	287 (4.0)	153 (3.1)	134 (6.2)	
	Peripheral vascular disease or abdominal aortic aneurysm	No	6,610 (92.4)	4,693 (93.8)	1917 (89.1)	<0.001
		Yes	546 (7.6)	312 (6.2)	234 (10.9)	
	Hypertension	No	4,693 (65.6)	3,532 (70.6)	1,161 (54.0)	<0.001
		Yes	2,463 (34.4)	1,473 (29.4)	990 (46.0)	
	Cerebrovascular disease	No	7,001 (97.8)	4,935 (98.6)	2066 (96.0)	<0.001
		Yes	155 (2.2)	70 (1.4)	85 (4.0)	
	Hemiplegia paraplegia or paralytic syndrome	No	6,967 (97.4)	4,868 (97.3)	2099 (97.6)	0.488
		Yes	189 (2.6)	137 (2.7)	52 (2.4)	
	Chronic obstructive pulmonary disease	No	6,331 (88.5)	4,506 (90.0)	1825 (84.8)	<0.001
		Yes	825 (11.5)	499 (10.0)	326 (15.2)	
	Diabetes	No	6,039 (84.4)	4,377 (87.5)	1,662 (77.3)	<0.001
		Yes	1,117 (15.6)	628 (12.5)	489 (22.7)	
	Cancer (including lymphoma and leukemia)	No	5,335 (74.6)	3,554 (71.0)	1781 (82.8)	<0.001
		Yes	1821 (25.4)	1,451 (29.0)	370 (17.2)	
	Childpugh grade	A	1,221 (73.9)	979 (81.2)	242 (54.0)	<0.001
		B	388 (23.5)	205 (17.0)	183 (40.8)	
		C	44 (2.7)	21 (1.7)	23 (5.1)	
	Preoperative anemia	No	3,735 (52.4)	3,044 (61.0)	691 (32.3)	<0.001
		Yes	3,398 (47.6)	1950 (39.0)	1,448 (67.7)	
	Preoperative increased creatinine	No	5,908 (85.6)	4,553 (93.7)	1,355 (66.4)	<0.001
		Yes	991 (14.4)	306 (6.3)	685 (33.6)	
	Preoperative leukocytosis (n[%])	No	5,857 (82.1)	4,269 (85.5)	1,588 (74.2)	<0.001
		Yes	1,276 (17.9)	725 (14.5)	551 (25.8)	
Emergency case (n[%])	Emergency		2025 (28.3)	1,161 (23.2)	864 (40.2)	<0.001
	Elective		5,131 (71.7)	3,844 (76.8)	1,287 (59.8)	
Surgical category (n[%])	Thoracic		608 (8.5)	539 (10.8)	69 (3.2)	0.589
	Vascular		536 (7.5)	380 (7.6)	156 (7.3)	
	General		1,639 (22.9)	1,091 (21.8)	548 (25.5)	
	Neurosurgery		1,369 (19.1)	972 (19.4)	397 (18.5)	
	Orthopedic		1,524 (21.3)	1,065 (21.3)	459 (21.3)	
	Other		1,480 (20.7)	958 (19.1)	522 (24.3)	
General anaesthesia (n[%])		Yes	6,701 (94.2)	4,814 (96.5)	1887 (88.7)	<0.001
		No	414 (5.8)	174 (3.5)	240 (11.3)	
Intraoperative hypotension (n[%])	MAP<55 mmHg at any time	No	6,117 (85.5)	4,400 (87.9)	1717 (79.8)	<0.001
		Yes	1,039 (14.5)	605 (12.1)	434 (20.2)	
Intraoperative mean heart rate (median[IQR])			71.80 [64.64,81.89]	70.44 [64.00,78.90]	76.53 [66.70,89.83]	<0.001
Intraoperative transfusion (n[%])		No	6,123 (85.6)	4,461 (89.1)	1,662 (77.3)	<0.001
		Yes	1,033 (14.4)	544 (10.9)	489 (22.7)	
Intraoperative blood loss (n[%])		<=400 mL	4,663 (65.2)	3,209 (64.1)	697 (32.4)	0.005
		>400 mL	2,493 (34.8)	1796 (35.9)	1,177 (54.7)	
Duration of surgery (n[%])		<=120 min	3,680 (51.4)	2,503 (50.0)	1,177 (54.7)	<0.001
		>120 min	3,476 (48.6)	2,502 (50.0)	974 (45.3)	
Preoperative laboratory tests	Hb (median[IQR])		126.00 [106.00,140.00]	129.00 [114.00,142.00]	112.00 [91.00,131.00]	<0.001
	WBC (median[IQR])		6.77 [5.23,9.57]	6.49 [5.11,8.89]	7.69 [5.70,11.14]	<0.001
	NLR (median[IQR])		3.56 [2.11,7.56]	3.03 [1.93,6.09]	5.32 [2.93,11.10]	<0.001
	BUN (median[IQR])		5.20 [4.10,6.70]	4.90 [3.90,6.00]	6.20 [4.60,9.10]	<0.001
	CRE (median[IQR])		70.00 [58.00,86.00]	68.00 [57.00,80.00]	81.00 [63.00,115.00]	<0.001
	GFR(median[IQR])		91.54 [75.78,104.72]	94.14 [82.54,106.29]	78.78 [50.65,99.23]	<0.001
	TBil (median[IQR])		11.70 [8.45,16.25]	11.70 [8.70,15.95]	11.70 [7.80,17.20]	0.487
	ALB (median[IQR])		40.40 [35.90,43.70]	41.30 [37.70,44.30]	37.10 [32.58,41.40]	<0.001
	ALT (median[IQR])		19.00 [13.00,31.00]	19.00 [13.00,30.00]	19.00 [12.00,34.00]	0.653
	LDH (median[IQR])		176.00 [150.00,216.00]	169.00 [146.00,200.00]	200.00 [166.00,263.00]	<0.001
	ALP (median[IQR])		77.00 [62.00,98.00]	77.00 [62.00,95.00]	79.00 [62.00,104.00]	0.001
	G (median[IQR])		5.70 [4.93,7.31]	5.53 [4.87,6.90]	6.34 [5.15,8.52]	<0.001
Postoperative outcomes	ICU admission(*n*[%])	No	5,057 (70.7)	3,719 (74.3)	1,338 (62.2)	<0.001
		Yes	2099 (29.3)	1,286 (25.7)	813 (37.8)	
	MINS (*n*[%])	No	6,495 (91.0)	4,760 (95.4)	1735 (80.8)	<0.001
		Yes	644 (9.0)	231 (4.6)	413 (19.2)	
	Length of hospital stay (median [IQR])		12.00 [8.00,20.00]	11.00 [8.00,18.00]	15.00 [9.00,25.00]	<0.001
	Death within 30 days (*n*[%])	No	6,157 (93.8)	4,489 (96.5)	1,668 (87.1)	<0.001
		Yes	410 (6.2)	162 (3.5)	248 (12.9)	
	Death within 90 days (*n*[%])	No	6,074 (92.5)	4,460 (95.9)	1,614 (84.2)	<0.001
		Yes	493 (7.5)	191 (4.1)	302 (15.8)	
	Death within 1 year (*n*[%])	No	6,539 (92.9)	4,767 (96.1)	1772 (85.2)	<0.001
		Yes	500 (7.1)	192 (3.9)	308 (14.8)	
	Death during follow-up (*n*[%])	No	6,067 (90.8)	4,459 (94.9)	1,608 (80.9)	<0.001
		Yes	617 (9.2)	238 (5.1)	379 (19.1)	

BMI, Body mass index; ASA-PS, American Society of Anesthesiologists Physical Status; CCI, Charlson Comorbidity Index; SORT, Surgical Outcome Risk Tool; RCRI, Revised Cardiac Risk Index; Hb, Hemoglobin; WBC, White blood cells; NLR, Neutrophil to lymphocyte ratio; BUN, Blood urea nitrogen; CRE, Creatinine; eGFR, Estimated glomerular filtration rate; TBil, Total bilirubin; ALB, Albumin; ALT, Alanine transaminase; LDH, Lactate dehydrogenase; ALP, Alkaline phosphatase; G, Blood glucose.

**Figure 1:**
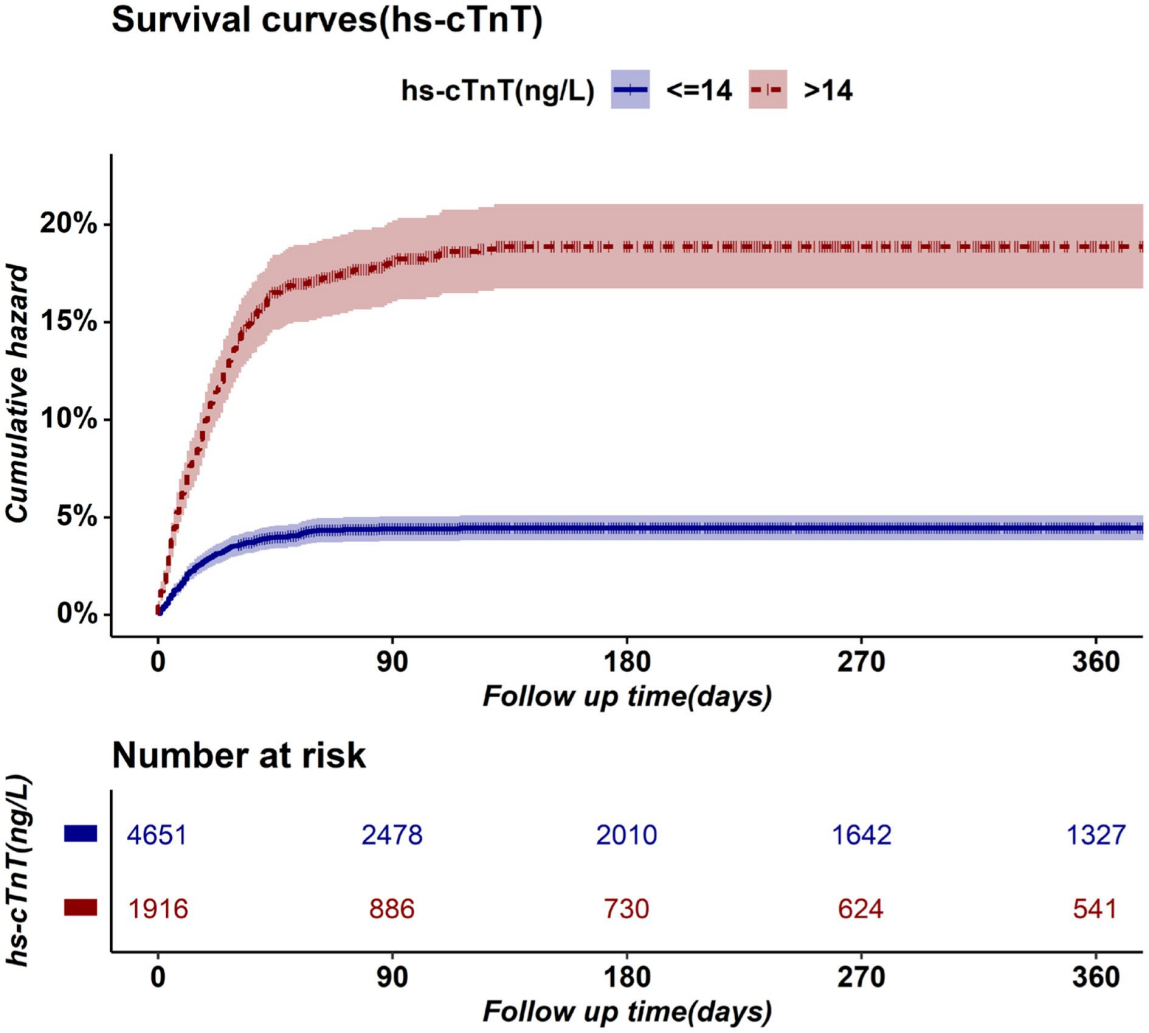
Kaplan–Meier Survival Plot for postoperative mortality.

**Figure 2:**
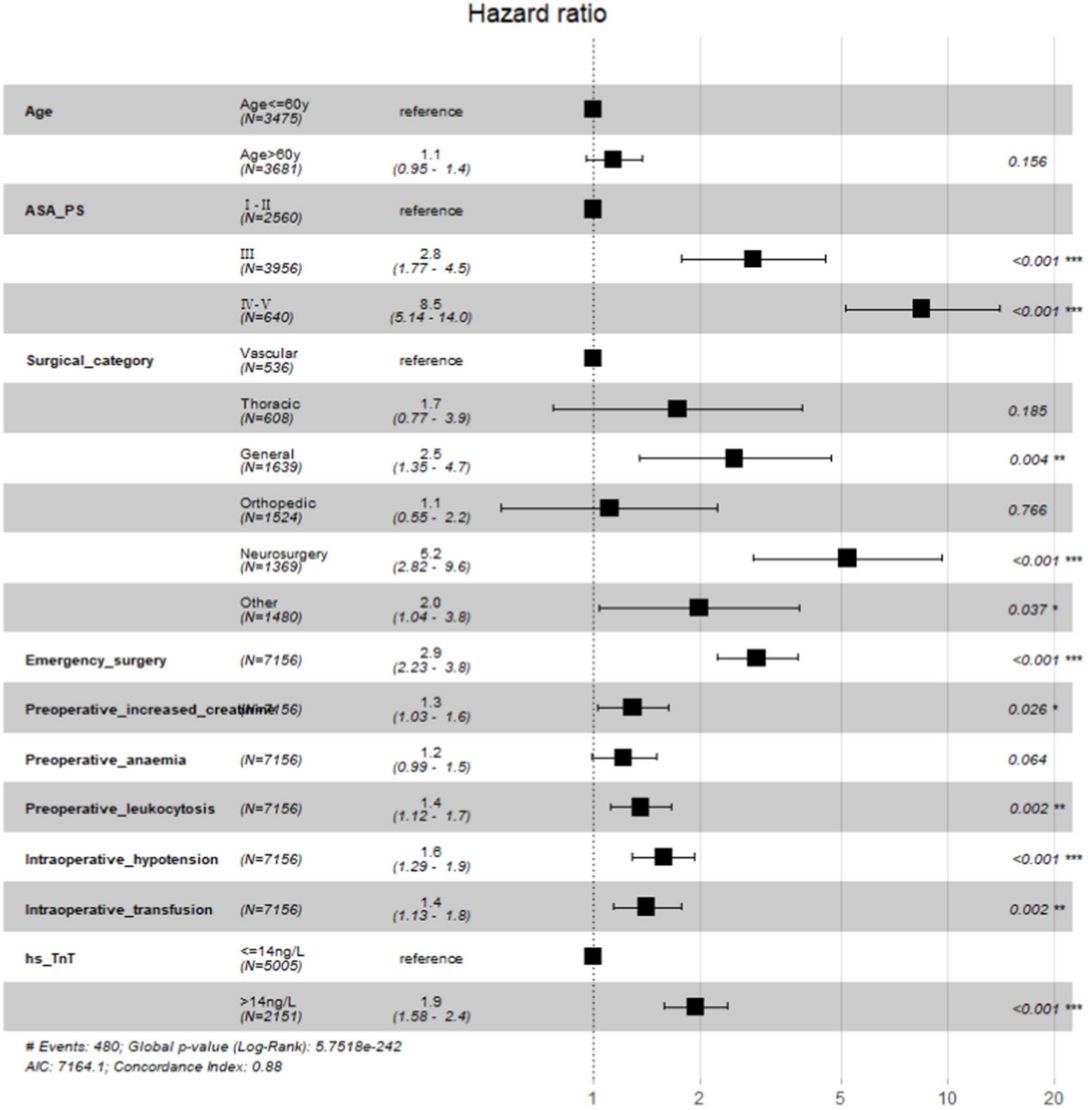
Forestplot of multivariable Cox Regression Model to Estimate one-year mortality.

Preoperative elevated hs-cTnT was associated with higher mortality in unadjusted survival analysis (*p* < 0.001 by log-rank test) (Figure 1). The hazard ratio of 30-day mortality was 3.90 (95% CI, 3.20–4.75; *p* < 0.001). It steadily increased and maintained statistical significance over the one-year period after surgery (HR at 1 year, 4.13; 95% CI, 3.45–4.95; *p* < 0.001). It’s worth noting that most events occurred within 3 months after surgery, afterwards the Kaplan–Meier plots did not seperate further. Figure 2 summarizes the multivariable Cox regression risk-adjustment model. The area under the ROC curve of the model was 0.88. The ROC and calibration curves were shown in Supplementary Figure 2. Results were similar in a multivariable Cox regression model that adjusted for all covariates in Table 2 (adjusted HR at 1 year, 1.93; 95% CI, 1.58–2.36; *p* < 0.001; Table 2).

**Table 2:** HRs for associations of hs-cTnT with outcomes.

Primary composite outcome	HR (95% CI)	aHR(95% CI)
30-day mortality	3.90 (3.20–4.75)	1.74 (1.39–2.18)
90-day mortality	4.07 (3.40–4.88)	1.89 (1.54–2.31)
1-year mortality	4.13 (3.45–4.95)	1.93 (1.58–2.36)
Secondary Outcomes	OR(95% CI)	aOR(95% CI)
ICU admission	1.76 (1.58–1.96)	1.52 (1.31–1.76)
MINS	4.91 (4.14–5.81)	3.01 (2.46–3.68)
Length of hospital stay	1.84 (1.68–2.01)	1.48 (1.34–1.64)

HR, Hazard ratio; aHR, Hazard ratio adjusted for all variables on Figure 2.

Preoperative elevated hs-cTnT was also associated with MINS, ICU admission, and LOS (*p* < 0.001 by Wald test for each outcome). In a univariable logistic regression model, ORs for associations of elevated hs-cTnT with MINS, ICU admission, and LOS were significant (MINS: odds ratio[OR] 4.91; 95% CI, 4.14–5.81, *p* < 0.001; LOS:OR 1.84; 95% CI, 1.68–2.01, *p* < 0.001; ICU admission: OR 1.76; 95%CI, 1.58–1.96, *p* < 0.001). Results were similar in a multivariable model adjusting for patient characteristics during the initial admission (ie, adjusted for all covariates in Table 2), with a significant association of with higher incidence of MINS, ICU admission, and LOS (MINS: adjusted odds ratio[aOR] 3.01; 95% CI, 2.46–3.69; *p* < 0.001; LOS: aOR 1.48, 95%CI 1.34–1.641; *p* < 0.001; ICU admission: aOR 1.52, 95%CI 1.31–1.76; *p* < 0.001).

### Subgroup analyses

**Figure 3:**
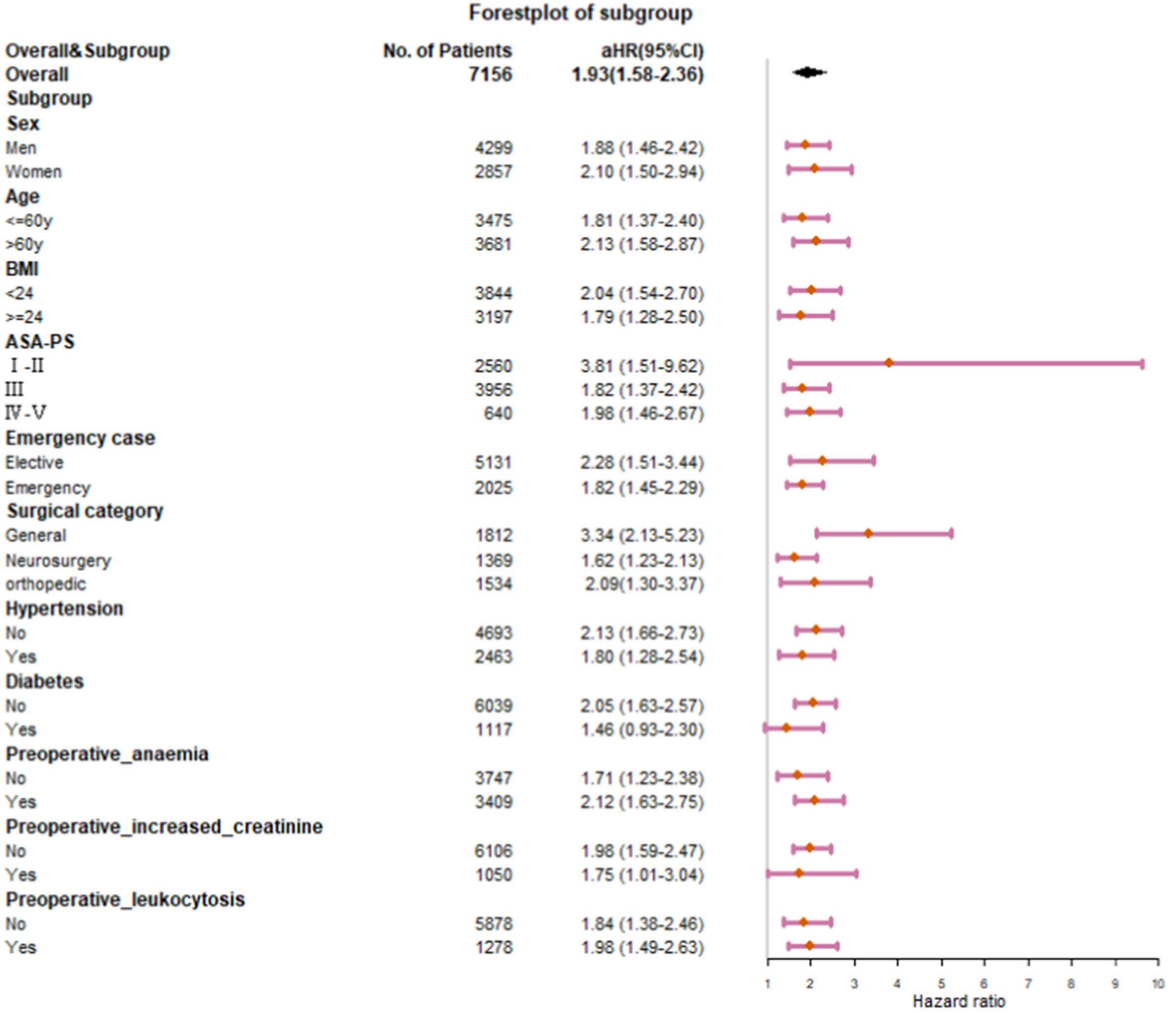
Association of elevated hs-cTnT with one-year mortality by Baseline Subgroups.

In subgroup analyses stratified by sex, age, BMI, ASA grades, surgical categories, degrees of surgical urgency, and preoperative comorbidities, the associations between preoperative hs-cTnT and one-year mortality remained in all categories (Figure 3). Subgroup analysis of diabetic patients showed insignificant results due to the small sample size, but the trend was consistent with the overall. In a subgroup analysis, preoperative hs-cTnT was most strongly associated with mortality among patients after general surgery (aHR, 3.34; 95% CI, 2.13–5.23; *p* < 0.001). In a subgroup analysis by ASA scores, preoperative hs-cTnT was most strongly associated with adverse outcomes among patients with ASA GradeI-II, followed by those with Grade III and IV-V (GradeI-II: aHR, 3.81; 95% CI, 1.51–9.62; *p* < 0.001; GradeIII: aHR, 1.82; 95%CI, 1.37–2.42; *p* < 0.001; GradeIV-V: aHR, 1.98; 95% CI, 1.46–2.67; *p* < 0.001). After adjusting for multiple factors, the results of a subgroup analysis suggest that preoperative hs-cTnT remains significantly correlated with mortality in patients who have undergone either elective surgery (aHR 2.28; 95% CI 1.51–3.44; *p* < 0.001) or emergency surgery (aHR 1.82; 95% CI 1.45–2.29; *p* < 0.001).

aHRs of other subgroups remained stable between 1.71 and 2.13.

### Mediation analyses

**Figure 4:**
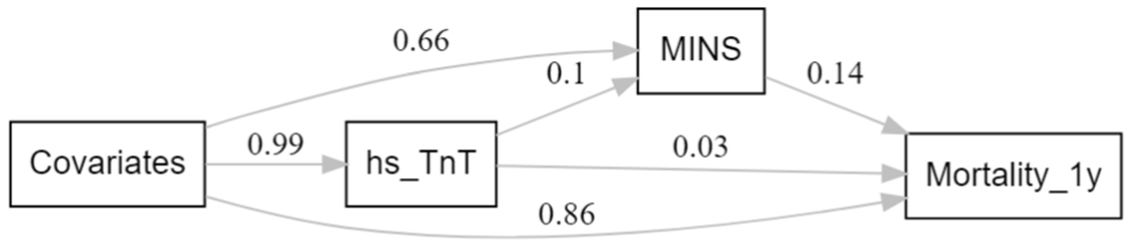
Simplified Structural equation modeling examining MINS as mediator between preoperative hs-cTnT and one-year mortality.

The SEM analysis showed that a significant main effect was found for elevated hs-cTnT with one-year mortality after surgery (*β* = 0.043, 95%CI, 0.037–0.049; *p* < 0.001). The natural direct effects (NDE) of TnT indicated that we would, on average, observe a 0.029 point (95% CI, 0.028–0.030; *p* < 0.001) increase in mortality if all study participants were free of MINS. The natural indirect effects (NIE) via MINS implied that we would, observe a 0.014 point (95% CI, 0.013–0.015; *p* < 0.001) increase in mortality among participants with elevated TnT. The proportion of the association between TnT and mortality mediated by MINS was 33.6% (95%CI 28.1–39.1%). Figure 4 illustrates a simplified SEM representation of the mediation effect of MINS.

## Discussion

In this large cohort of patients with the preoperative hs-cTnT test, we observed that preoperative elevated hs-cTnT was persistently associated with a higher risk of death for at least 1 year. Cox regression models in different periods showed the associations remained significant and substantial throughout the follow-up period and gradually increased over time. This indicates that elevated hs-cTnT before surgery was associated with long-term outcomes and suggests that prior studies underestimate the harms of elevated hs-cTnT by only assessing short-term outcomes.

Only a few studies (3, 11–15) have examined the long-term risk of death in patients with elevated cTn before noncardiac surgery. Among them, only Nagele and colleagues (3) analyzed the correlation between preoperative hs-cTnT and long-term mortality. However, intraoperative anesthesia management, surgery-related factors, and RCRI score, which have been shown to have a significant impact on postoperative myocardial injury and death, were also ignored (3, 34, 35). The sensitivity and accuracy of the Cox proportional hazard model have also not been reported, so the conclusion may be controversial. In our study, the proportion of patients with preoperative high troponin and postoperative death was lower than in previous prospective studies. This may be due to the increasing attention of clinicians to the prognostic value of troponin in recent years, and more and more patients will be required to have troponin tests before surgery to facilitate the comparison of results before and after surgery. Due to the retrospective nature of this study, clear criteria for pre-surgical hs-cTnT testing cannot be established, as in randomized controlled trials. Clinical physicians typically rely on their clinical experience and guidelines to determine whether to perform pre-surgical hs-cTnT testing, which may include recent myocardial infarction, recent acute myocardial injury, known coronary artery disease, known heart failure, and symptoms of angina. However, this is precisely the feature encountered in real-world big data analysis, where actual data often integrates a multitude of complex situations, and their conclusions more intuitively reflect clinical scenarios and adapt to the complexity of the real world. In this study, it was evident that more patients who were generally considered to be at low or intermediate risk underwent troponin testing before surgery. Our Cox proportional hazard model did not include RCRI scores as the former studies did. However, our model was the best model (AUC = 88%) selected by stepwise regression after including a large number of confounding factors (including RCRI and the other four surgical prediction models). After the variable screening, the ASA score remained in the final model while RCRI was screened out, which may be explained by the poor performance of RCRI in predicting death (17).

Subgroup analysis showed a stronger association in patients with lower ASA scores, which may suggest that hs-cTnT may have a stronger application prospect in patients at low and moderate risk. Preoperative hs-cTnT was most strongly associated with mortality among patients after general surgery. It should be noted that this study should exclude patients who have experienced acute coronary syndrome (ACS) recently or have developed heart failure within 1 month before surgery. However, in reality, at our hospital, patients who have experienced ACS or heart failure within 1 month prior to surgery are usually not scheduled for non-cardiac surgery, so we did not specifically exclude them. Nevertheless, we conducted the subgroup analysis on elective surgery to exclude all emergency or urgent cases, thereby reducing the likelihood of patients experiencing recent ACS. The results were further confirmed by the subgroup analysis. Further studies in patients of relevant subspecialty are needed in the future. Mediation analyses indicated a 33.6% mediation effect of MINS between hs-cTnT and one-year mortality. This finding reinforces the value of preoperative hs-cTnT detection and contributed to a better understanding of the relation between preoperative hs-cTnT, preoperative change in hs-cTnT, and mortality.

Several limitations need consideration. As in any observational analysis, unobserved confounding may distort our results (36). A critical assumption of mediation analysis is that of no exposure-induced mediator-outcome confounding, and any violation of this assumption would have implications for the identification of natural associations. Another limitation is that censored death data during follow-up may not be random. Multiple data sources need to be adopted to attenuate misclassification bias in the future. In addition, the study sample only included only patients who underwent preoperative tests, which may limit the generalizability of the study findings. Due to the retrospective nature of this study, it is not possible to accurately determine the preoperative hs-cTnT testing standards. However, it is precisely because of this that the results of the study can reflect the complex situation in clinical practice. Our results suggest that preoperative hs-cTnT is an additional potentially modifiable risk for MINS and all-cause mortality, especially in patients with low ASA status who are easily overlooked. However, the extent to which the relationship is causal and thus amenable to intervention remains unknown. This study mainly focuses on the effect of preoperative hs-cTnT on prognosis, in order to help clinicians get effective information before surgery. The changes of hs-cTnT before and after surgery and the level of hs-cTnT after surgery may need more prospective experiments in the future.

## Conclusion

In summary, preoperative hs-cTnT over 14 ng/L has a persistent and significant association with a significant increase in long-term mortality, MINS, LOS, and ICU admission. MINS explained approximately 33.6% of the effect on mortality due to preoperative hs-cTnT levels.

## Data availability statement

The datasets presented in this article are not readily available because the hospital ethics committee requested that the data not be made public Requests to access the datasets should be directed to liubinhxyy@163.com.

## Ethics statement

The studies involving human participants were reviewed and approved by Ethics Committee of Sichuan University (Project No. 1082 in 2021). Written informed consent from the participants’ legal guardian/next of kin was not required to participate in this study in accordance with the national legislation and the institutional requirements.

## Author contributions

All authors had full access to the data in the study and take responsibility for the integrity of the data and the accuracy of the data analysis. YCZ: data analysis and interpretation, prepared the draft manuscript, revised the draft manuscript, and approved the final manuscript for submission. YDB: data acquisition and curation, data analysis and interpretation, revised the draft manuscript. QY: follow-up data collection, ethical approval and trial registration. BL: designed and conceptualized the study, funding acquisition, data acquisition and curation, data analysis and interpretation, revised the draft manuscript. All of the authors agree to be accountable for all aspects of the work and in ensuring that questions related to the accuracy or integrity of any part of the work are appropriately investigated and resolved.

## Funding

This work was supported by the department fund of Natural Science Foundation of Sichuan Province (No. 2022NSFSC1297) and the Post-Doctor Research Project of West China Hospital of Sichuan University (No. 2021HXBH079). The funding sources had no role in the design of this study and the analysis and interpretation of the results.

## Conflict of interest

The authors declare that they have no known competing financial interests or personal relationships that could have appeared to influence the work reported in this paper.

## Publisher’s note

All claims expressed in this article are solely those of the authors and do not necessarily represent those of their affiliated organizations, or those of the publisher, the editors and the reviewers. Any product that may be evaluated in this article, or claim that may be made by its manufacturer, is not guaranteed or endorsed by the publisher.

## Figures and Tables

**Table:** 

hs-cTnT	High-sensitive cardiac troponin T
cTnI/T	Cardiac troponin I and T
MINS	Myocardial injury after noncardiac surgery
LOS	Length of hospital stay
aHR	Adjusted hazard ratio
aOR	Adjusted odds ratio
MACEs	Major adverse cardiac events
PMI	Postoperative myocardial injury
STROBE	Strengthening the Reporting of Observational Studies in Epidemiology
HIS	Hospital Information System
AIMS	Anesthesia Information Management System
ELISA	Elecsys Troponin T hs of non-competitive enzyme-linked immunosorbent assays
IQR	Inter-quartile range
VIF	Variance inflation factor
ROC	Receiver operating curve
CIs	Confidence intervals
SEM	Structural equation modeling
NDE	Natural direct effects
NIE	Natural indirect effects
URL	Upper range limit
BMI	Body mass index
ASA-PS	American Society of Anesthesiologists Physical Status
CCI	Charlson Comorbidity Index
SORT	Surgical Outcome Risk Tool
RCRI	Revised Cardiac Risk Index
Hb	Hemoglobin
WBC	White blood cells
NLR	Neutrophil to lymphocyte ratio
BUN	Blood urea nitrogen
CRE	Creatinine
eGFR	Estimated glomerular filtration rate
TBil	Total bilirubin
ALB	Albumin
ALT	Alanine transaminase
LDH	Lactate dehydrogenase
ALP	Alkaline phosphatase
G	Blood glucose
