# Supplementary material for: Assessment of the prognostic value of preoperative high-sensitive troponin T for myocardial injury and long-term mortality for groups at high risk for cardiovascular events following noncardiac surgery: a retrospective cohort study
Source: Front Med (Lausanne). 2023 Jun 22;10:1135786. doi: 10.3389/fmed.2023.1135786 (PMC10325788; doi:10.3389/fmed.2023.1135786)
Supplement: Supplementary file 1 [file Data_Sheet_1.docx]

**Supplement Figure 1. Study flowchart**


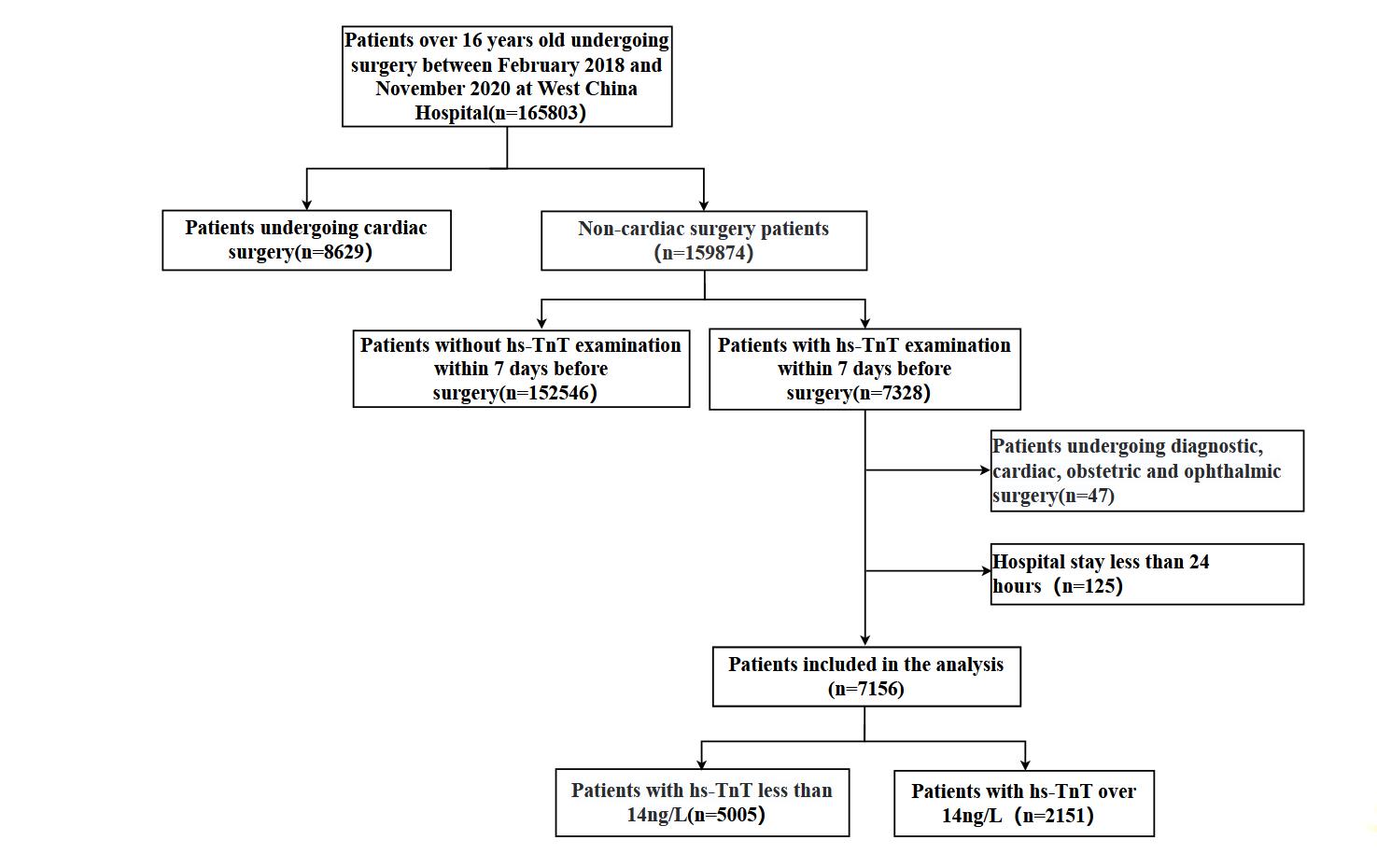


**Supplement Figure 2.** **ROC and calibration curves of multivariable Cox regression model**


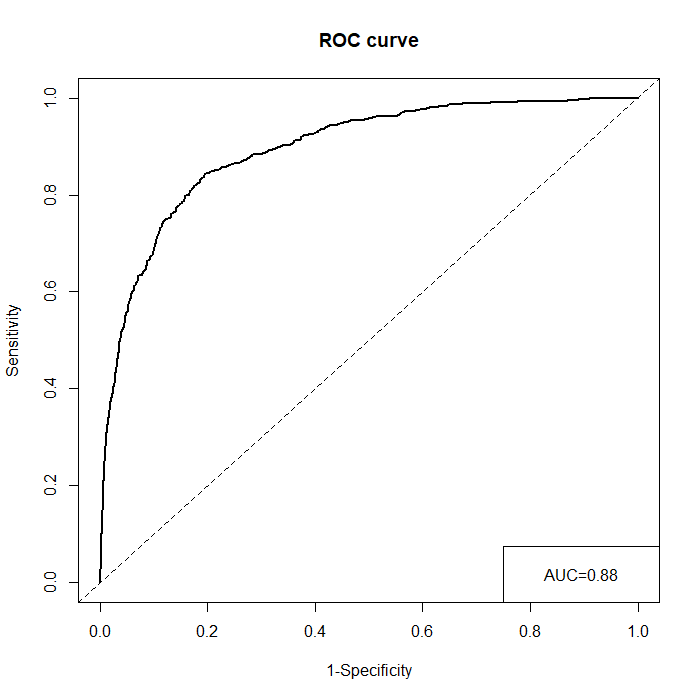
**
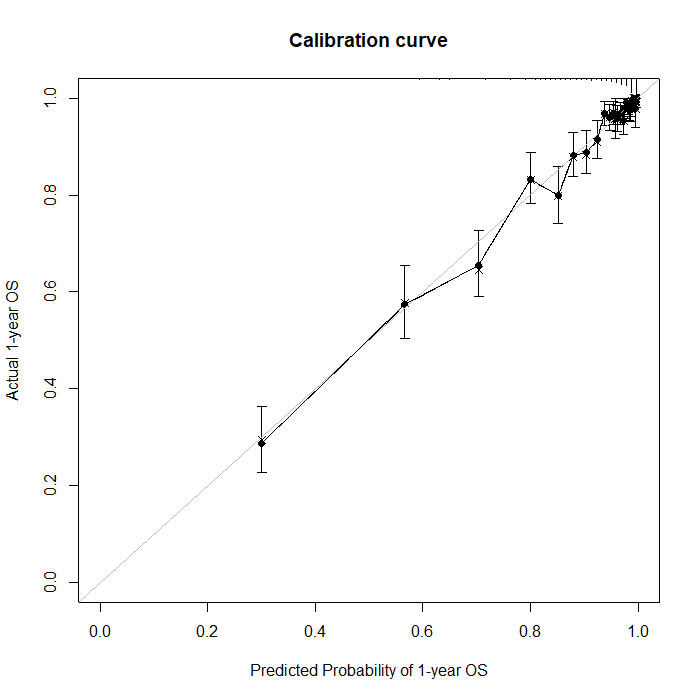
**

**Supplement table 1. Univariate analysis for each factor**

**Abbreviations:** BMI: Body mass index, ASA-PS: American Society of Anesthesiologists Physical Status, CCI: Charlson Comorbidity Index, SORT: Surgical Outcome Risk Tool, RCRI: Revised Cardiac Risk Index
